# Supplementary material for: Bioinspired “cage traps” for closed-loop lead management of perovskite solar cells under real-world contamination assessment
Source: Nat Commun. 2023 Aug 7;14:4730. doi: 10.1038/s41467-023-40421-8 (PMC10406821; doi:10.1038/s41467-023-40421-8)
Supplement: Supplementary file 1 — Supplementary Information [file 41467_2023_40421_MOESM1_ESM.pdf]

Supporting Information for

Bioinspired “cage traps ”for closed-loop lead management of perovskite solar cells under real-world contamination assessment

Huaiqing Luo[1,†], Pengwei Li[1,†], Junjie Ma[1,*], Xue Li[1], He Zhu[1], Yajie Cheng[1], Qin Li[1], Qun Xu[1], Yiqiang Zhang[1,*] & Yanlin Song[2,*]

[1] Henan Institute of Advanced Technology, College of Chemistry, Zhengzhou University, Zhengzhou 450001, P. R. China.

[2] Key Laboratory of Green Printing, Institute of Chemistry, Chinese Academy of Sciences, Beijing Engineering Research Center of Nanomaterials for Green Printing Technology, National Laboratory for Molecular Sciences, Beijing 100190, P. R. China.

* Corresponding authors: junjiema@zzu.edu.cn; yqzhang@zzu.edu.cn; ylsong@iccas.ac.cn

[†] Huaiqing Luo and Pengwei Li contributed equally to this work.

The Supplementary Information include:

Supplementary Note

Supplementary Figs. 1 to 31

Supplementary Table 1 to 5

Other Supplementary Information for this manuscript include the following:

Movie 1.

Table of Contents

1. Supplementary Note 3

2. Supplementary Figures 4

3. Supplementary Tables 25

4. Supplementary References...29

1. Supplementary Note

Supplementary Note 1 Mathematical models established based on Langmuir isothermal adsorption equation

The distribution coefficient (K_d) was calculated to explore the relative ratio of Pb concentration in the solid phase (MM, BCT) and liquid phase when adsorption reaches equilibrium. The K_d is defined as[1]:

$$K_d = \frac{Pb\ sorbed\ on\ sorbents(mg \cdot g^{-1})}{Pb\ in\ solution(mg \cdot mL^{-1})} = \frac{C_i - C_e}{C_e} \times \frac{V}{m} \qquad (1)$$

Where C_i is the initial concentration of the Pb ion, C_e is the final equilibrium concentration, V is the volume of the solution (mL) and m is the weight of the sorbent used (g).

Supplementary Note 2 Calculation process of adsorption capacity and pseudo-second-order kinetic

Adsorption capacity is one of the key indexes to quantitatively evaluate the adsorption effect of MM and BCT on Pb ion, which can be obtained by Equation (2):

$$q_t = \frac{(C_0 - C_t) \times V}{m} \qquad (2)$$

Where q_t is the amount of Pb adsorbed at moment t (mg/g), C_t is the residual Pb ion concentration at moment t (mg/L).

The Pb equilibrium adsorption capacity (q_e) of the MM and the BCT in the whole adsorption process are quantitatively determined as 14.22 and 32.934 mg/g, respectively. The q_e is defined as:

$$q_e = \frac{(C_0 - C_e) \times V}{m} \qquad (3)$$

Where q_e is the adsorption amount at adsorption equilibrium (mg/g).

To quantitatively depict adsorption kinetics of BCT and MM, the whole adsorption process was fitted via the pseudo-second-order mathematical model[2]:

$$\frac{dq_t}{dt} = k_2 \times (q_e - q_t)^2 \tag{4}$$

$$\frac{t}{q_t} = \frac{1}{k_2 \times {q_e}^2} + \frac{1}{q_e} \tag{5}$$

Supplementary Note 3 Calculation formula of heavy metal pollution assessment method

The detailed calculating formulas are defined as shown in Equations (6)-(8).

$$I_{geo} = Log_2\left(\frac{C_i}{K \times C_0}\right) \tag{6}$$

$$P_i = \left(\frac{C_i}{C_0}\right) \tag{7}$$

$$P_N = \sqrt[2]{\frac{P_{iave}^2 + P_{imax}^2}{2}} \tag{8}$$

Where C_i is the measured value of soil heavy metal content in the Yellow River; K is the constant factor usually with a value of 1.5 used to correct the potential variations in the background values associated with lithogenic effects; C_0 is the background value of soil pollutants; P_{iave} is the average value of P_i; P_{imax} is the maximum value of P_i.

2. Supplementary Figures

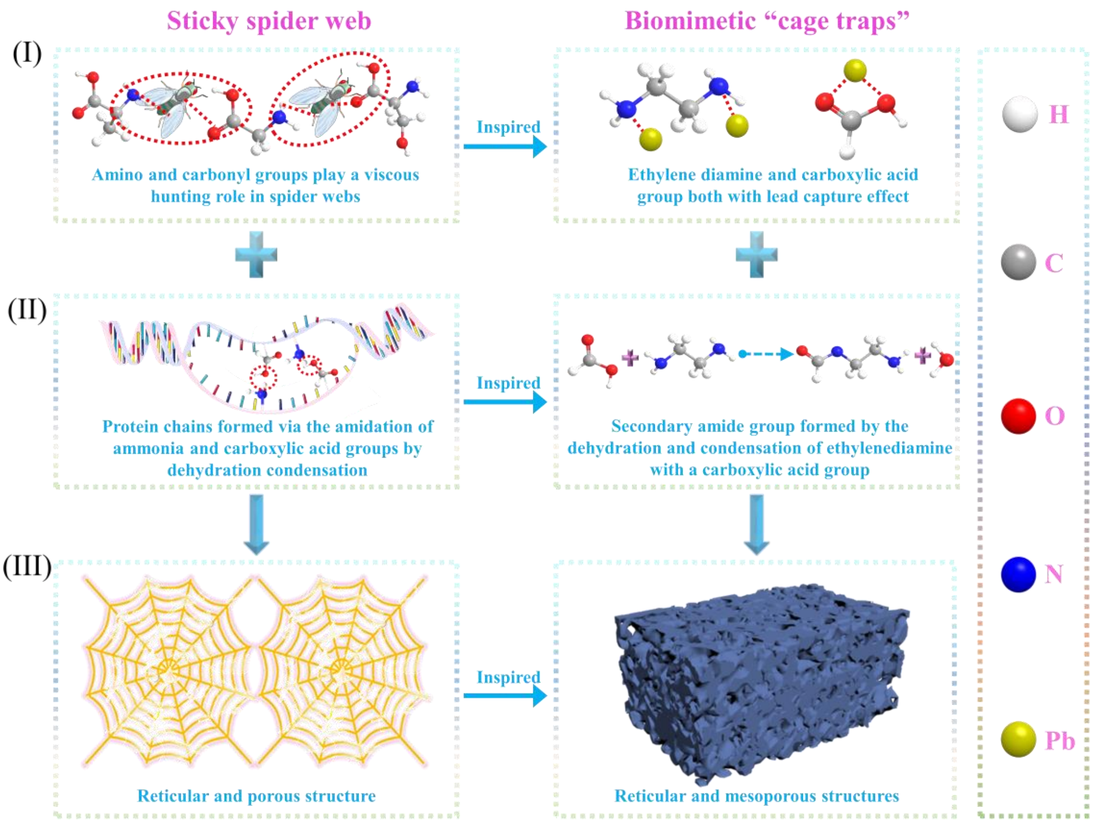

Supplementary Figure 1. Bionic synthesis mechanism of BCT based on spider web hunting mechanism. (I), Selection of Pb capture functional group inspired by adhesive properties of the spider mucilage. (II), Selection of CONH-R-NH_2 molecular synthetic mechanism inspired by the dehydration and condensation reaction of protein chains of spider web. (III), Selection of MM inspired by reticular and porous structure of spider web.

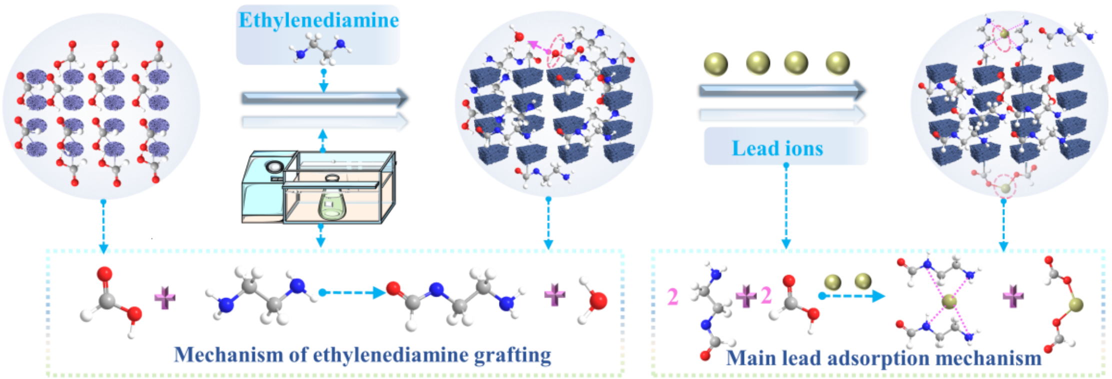

Supplementary Figure 2. The chemical synthesis route and Pb capture mechanism

of BCT. The mechanism of chemical synthesis of BCT and Pb chelation behavior of BCT. BCT was prepared based on ethylenediamine by dehydration condensation with -COOH group on MM to form -CONH-R-NH_2 group. The chemical chelation behavior of BCT to Pb was achieved via the bonding between -CONH-R-NH_2 group and Pb^{2+}.

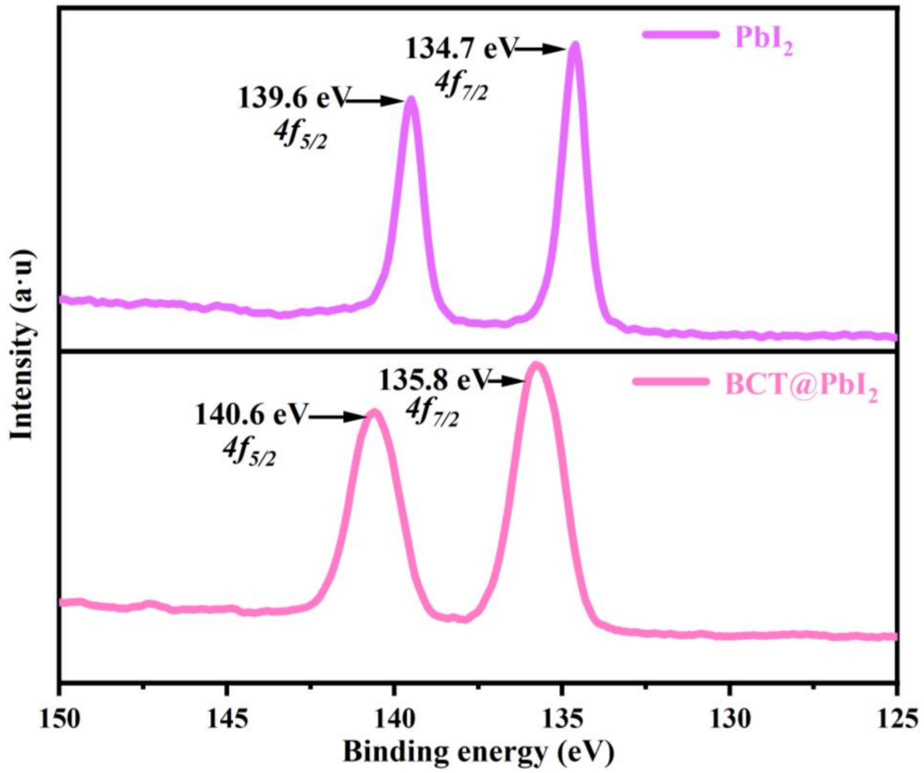

Supplementary Figure 3. XPS spectra of *Pb4f* for MM, BCT and PbI_2@BCT. The peaks corresponding to *Pb4f5/2* and *Pb4f7/2* shift from 139.6eV and 134.7eV to higher binding energies of 140.6 eV and 135.8 eV respectively for PbI_2@BCT samples.

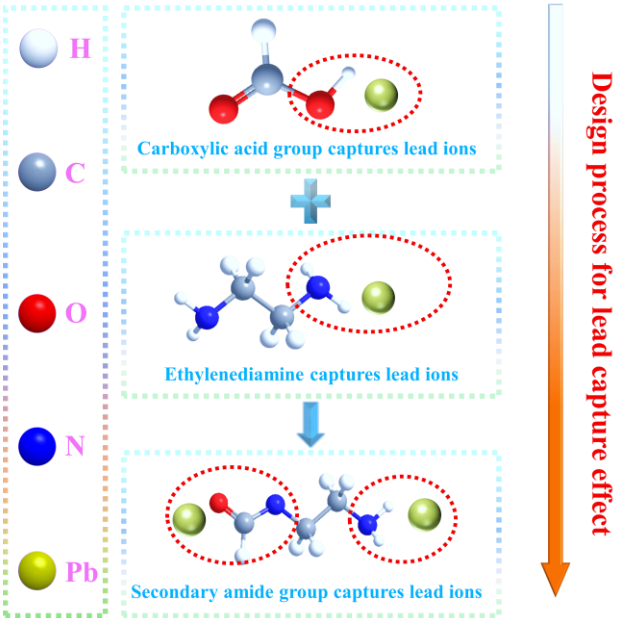

Supplementary Figure 4. Synergistic Pb adsorption effect of -CONH and -NH_2. -C=O group and -NH_2 group have strong coordination with Pb. When -COOH group is dehydrated and condensed with EDA to form CONH-R-NH_2, CONH-R-NH_2 has both -CO group and -NH_2 group, which can form synergistic Pb coordination.

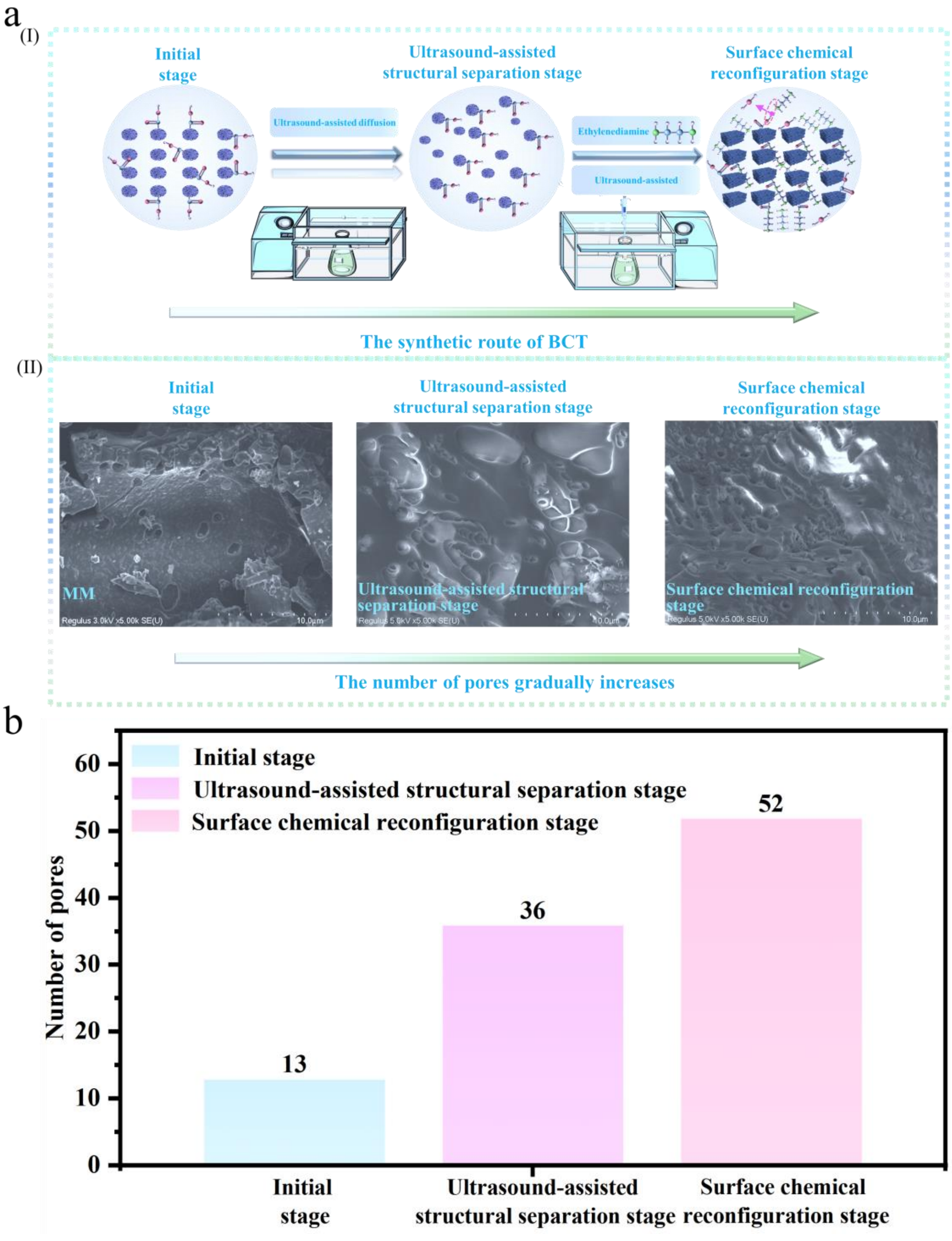

Supplementary Figure 5. The surface morphology characteristics of BCT in initial state, ultrasound-assisted structural separation stage and surface chemical reconfiguration stage during the synthesis of BCT. a, (I) (II)The SEM pictures of different stages in BCT synthesis route. b, The number of pores in different stages of BCT synthesis route.

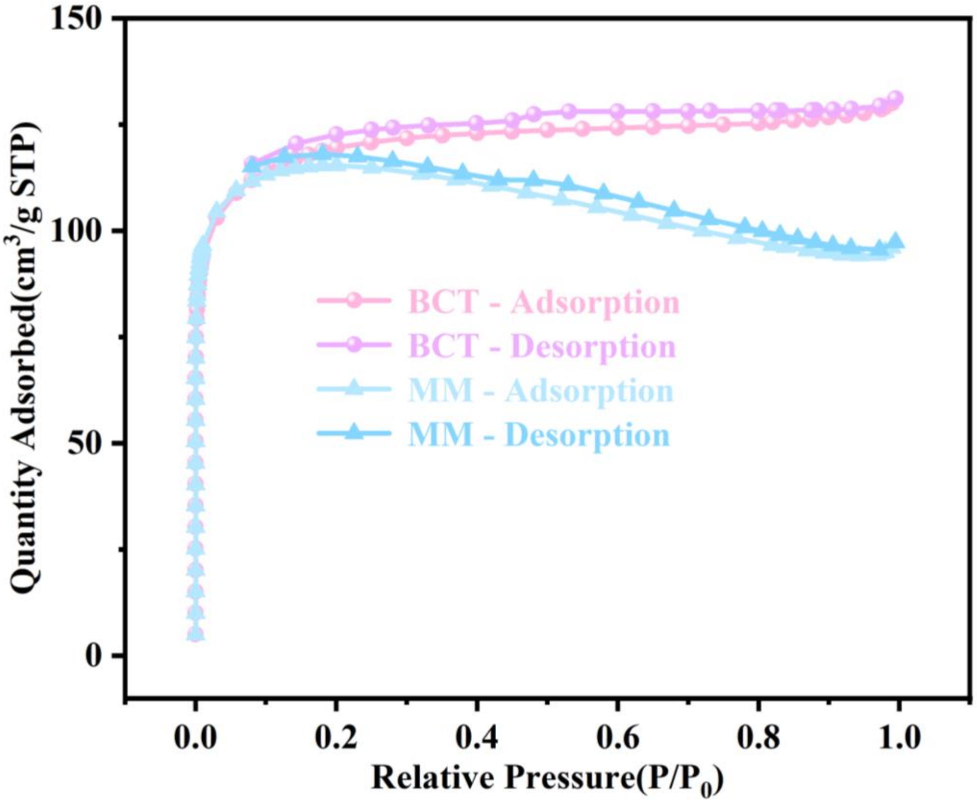

Supplementary Figure 6. Surface characterization of MM and BCT. Nitrogen adsorption-desorption isotherms of MM and BCT. The external surface area of BCT is 108.4398 $m^2\ g^{-1}$, which is more than twice as large as MM (40.2946 $m^2\ g^{-1}$).

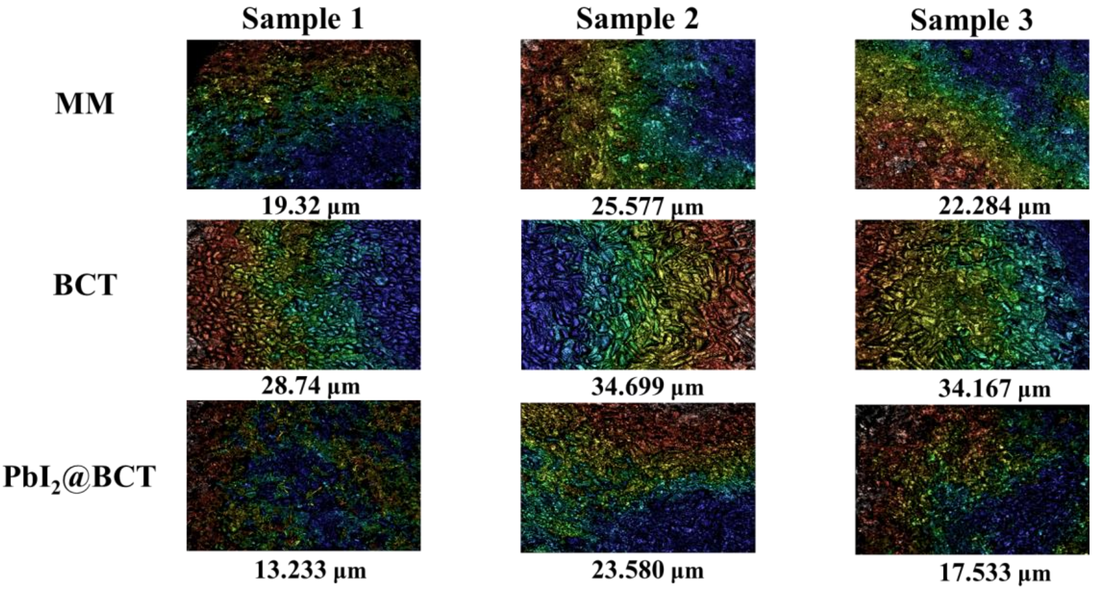

Supplementary Figure 7. 3D optical profilometer measurement on MM, BCT and PbI_2@BCT. The average surface roughness of MM was increased from 22.393µm to

32.535μm (BCT) due to the high-frequency ultrasonic vibration during the EDA self-assembly grafting process. When BCT adsorbed PbI_2, its average surface roughness decreased from 32.535μm to 18.115μm.

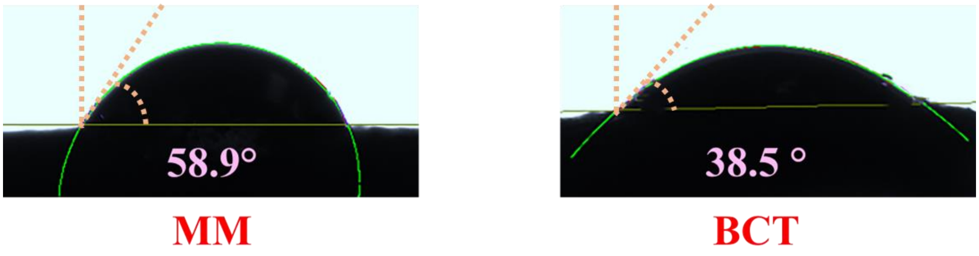

Supplementary Figure 8. The corresponding water contact angles of MM (58.9°) and BCT (38.5°) surface. BCT has better wettability than MM, which is more conducive for capillary adsorption

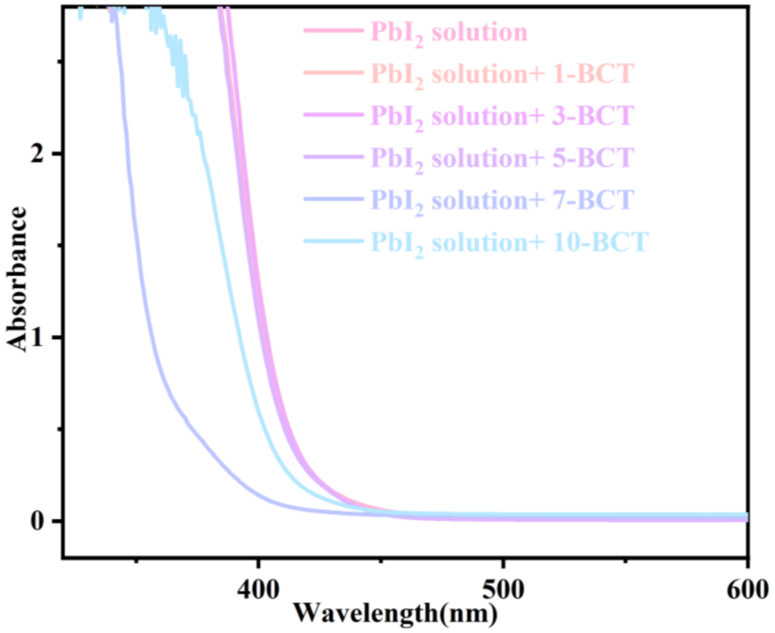

Supplementary Figure 9. The UV-visible absorption spectra of residual PbI_2 concentration in DMF. The absorbance of residual PbI_2 in the solution after adsorbing by MM self-assembled with 7 mL EDA shows the least UV absorbance.

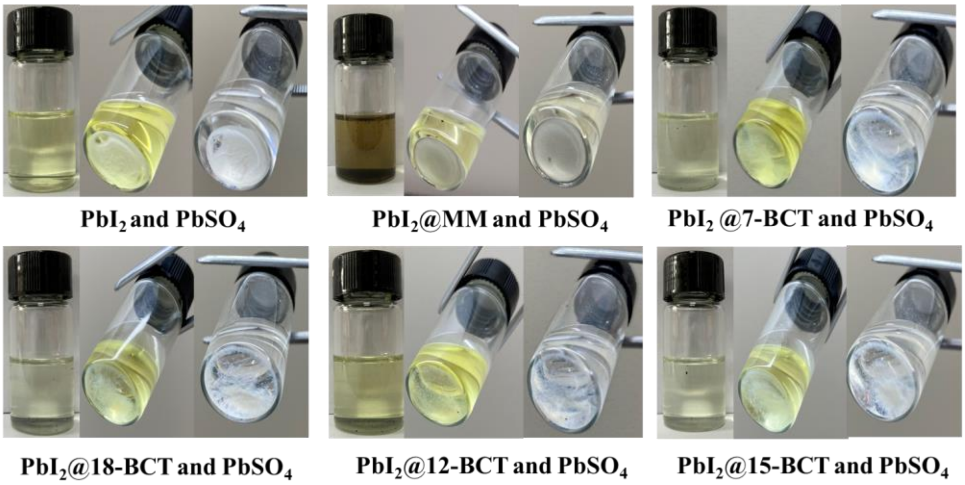

Supplementary Figure 10. Preliminary study on adsorption of PbI_2 by BCT. Photographs of $PbSO_4$ precipitation from PbI_2 solutions which were adsorbed by different types of BCT. The PbI_2 solution was adsorbed by MM with 7 mL EDA self-assembled, which has the minimum $PbSO_4$ precipitation.

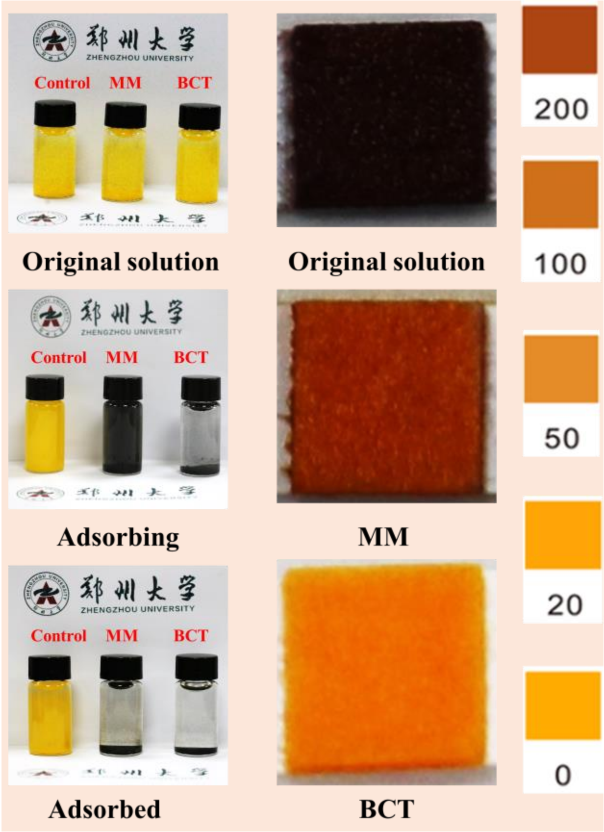

Supplementary Figure 11. The test results of Pb ion test paper. MM and BCT were immersed into the $FAPbI_3$ perovskite powders solutions. The residual Pb ion

concentration in $FAPbI_3$ perovskite powder solution via using Pb ion testing paper. The $FAPbI_3$ perovskite powder solution which was adsorbed by BCT shows the lowest Pb ions concentration (closing to ~ 0ppm), proving the effectiveness of BCT in Pb chelation.

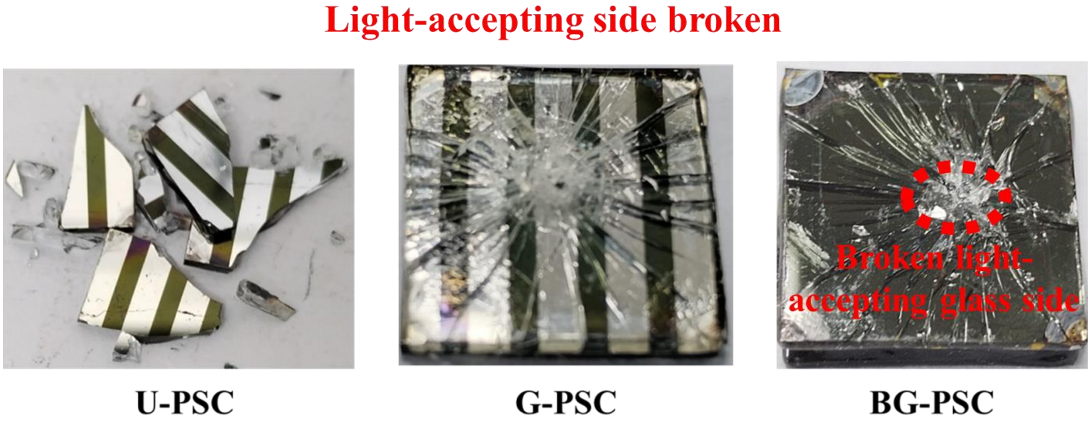

Supplementary Figure 12. **Typical photographs for the preparation of damaged U-PSC, G-PSC and BG-PSC for Pb leakage tests**. The damage levels and crack areas in the three groups of devices were controlled to be very similar in each hail simulation test.

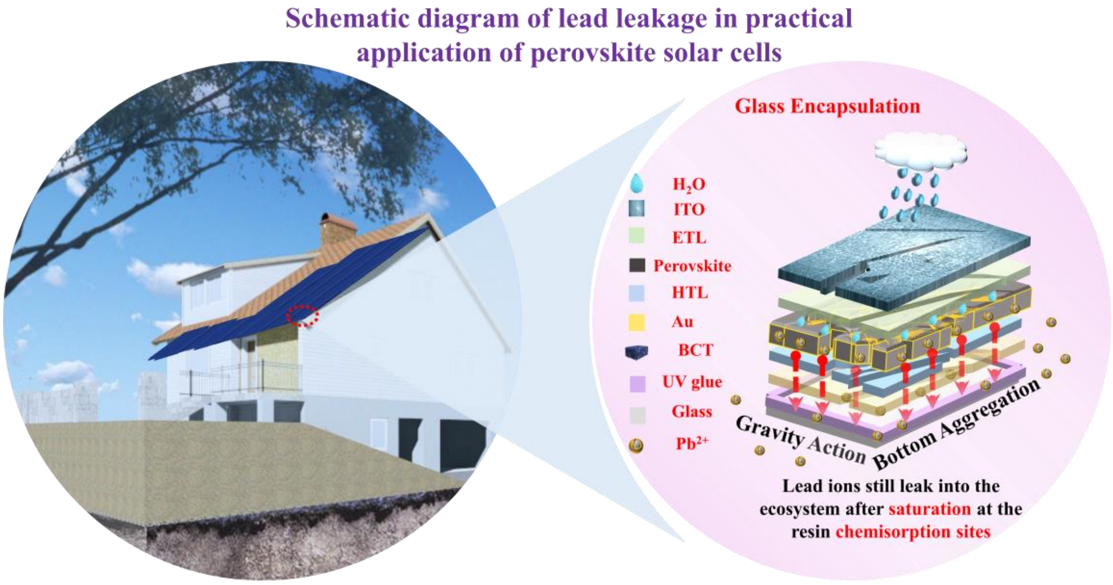

Supplementary Figure 13. **The Pb leakage process of glass-encapsulated PSCs in**

actual operation. The leaked Pb components tend to flow to the back side along the cracks within the damaged PSCs by gravity, because modules are installed at a large inclination angle to optimize sunlight exposure for that location.

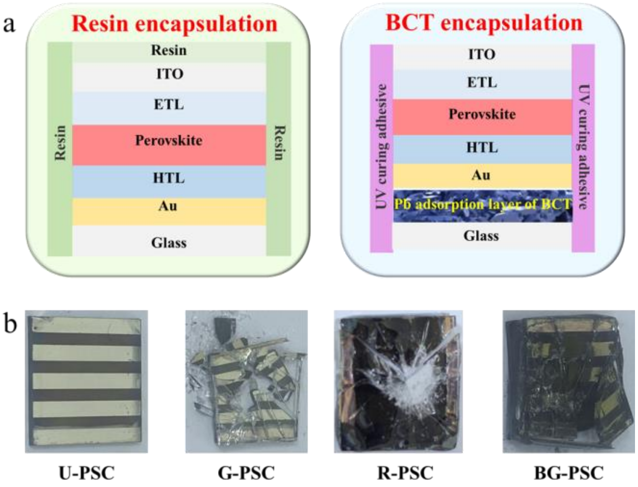

Supplementary Figure 14. The effect of different install position of functional materials on Pb leakage suppression performance under complete destructive conditions. a, The methods of encapsulating PSCs with resin and BCT. b, Typical photographs of completely cracked U-PSC, G-PSC, R-PSC and BG-PSC.

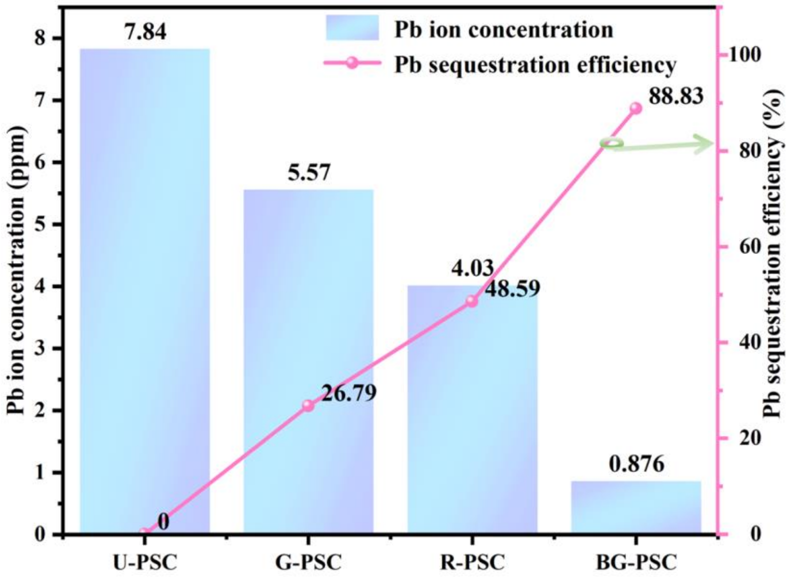

Supplementary Figure 15. The Pb leakage and Pb sequestration efficiencies of the completely cracked U-PSC, G-PSC, R-PSC and BG-PSC in DI water. The leaked

Pb ions are 7.84, 5.57, 4.03 ppm and 0.876 ppm for U-PSC, G-PSC, R-PSC and BG-PSC, respectively. U-PSC, G-PSC, R-PSC and BG-PSC exhibit the Pb sequestration efficiency of 0.00%, 26.79%, 48.59% and 88.83%, respectively.

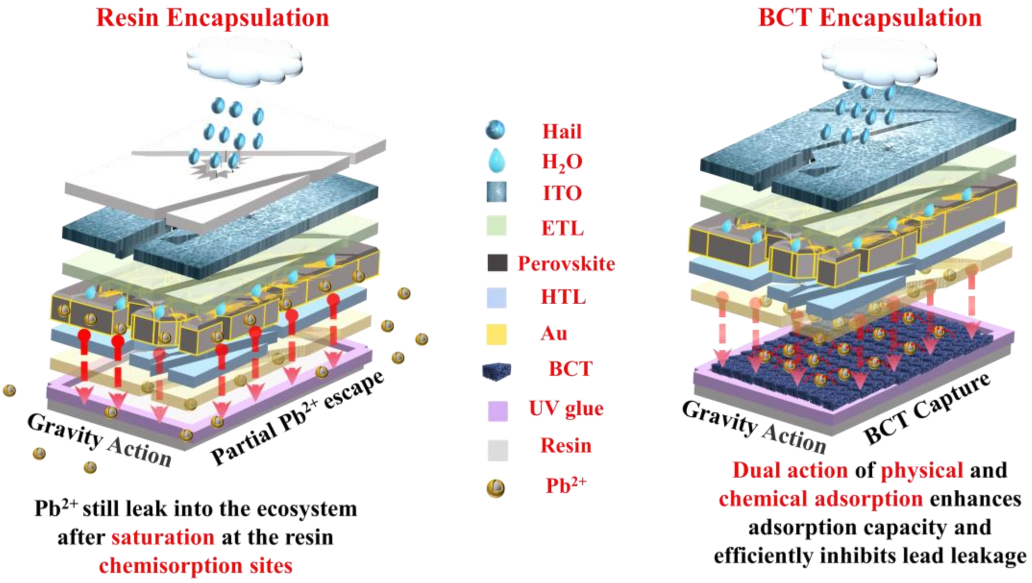

Supplementary Figure 16. The Pb leakage of PSCs encapsulated by semi-transparent Pb adsorbent materials and BCT during actual operation. The leaked Pb components tend to flow towards the backside of the device, and effective semi-transparent Pb adsorbents for capturing them in this location are still limited. However, BCT adsorbents with an unlimited thickness requirement are encapsulated on the backside of the device, which has resulted in superior Pb capture performance under ambient stimuli.

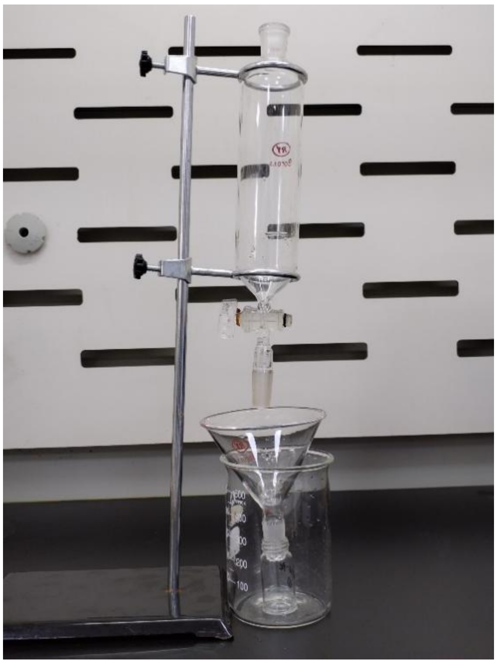

Supplementary Figure 17. The equipment for simulating acid rain dripping tests. The damaged U-PSC, G-PSC and BG-PSC are placed on the funnel. The nitric acid solution with a pH = 4.2 was titrated on the damaged panels at a flow rate of 10 mL/h for 1 h to simulate acid rain erosion. Then, the contaminated solution was collected in a bottle for ICP-OES test.

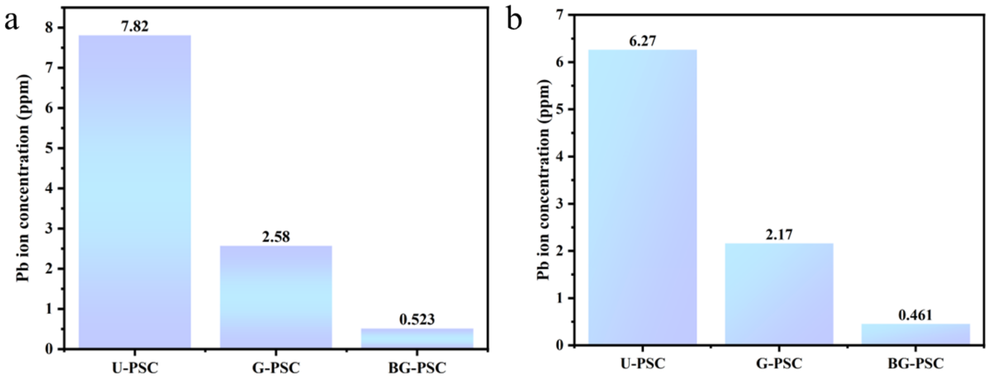

Supplementary Figure 18. The Pb leakage test results of crushing devices in alkaline environment. The Pb immobilization efficiency of U-PSC, G-PSC and BG-PSC at aqueous condition with pH=10(a) and pH=11(b).

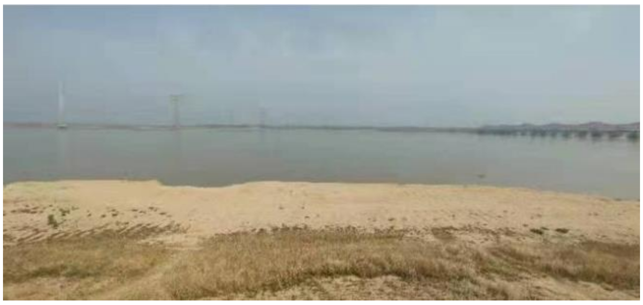

Supplementary Figure 19. The sampling position of real test environment. Photograph of sampling site of Yellow River water and Yellow River soil.

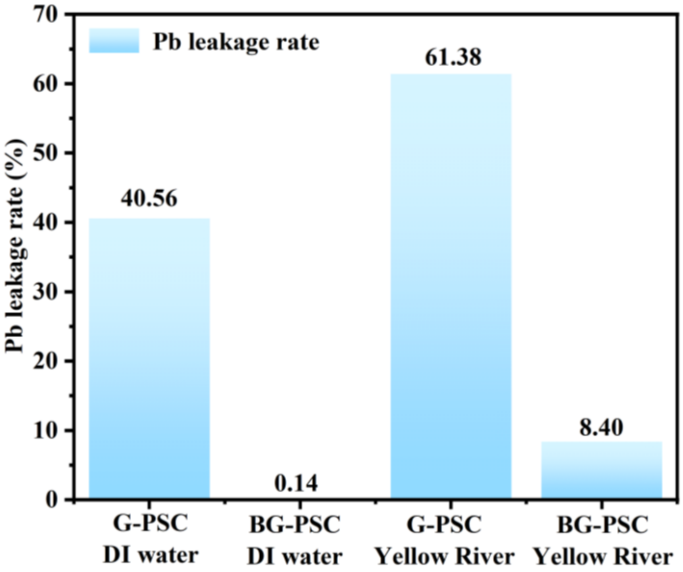

Supplementary Figure 20. Comparison of Pb leakage rates of G-PSC and BG-PSC in deionized water and Yellow River water. Compared with the Pb leakage rates of G-PSC and BG-PSC in DI water of 40.56% and 0.14%, their Pb leakage rates in the Yellow River water increased to 61.38% and 8.4%, respectively.

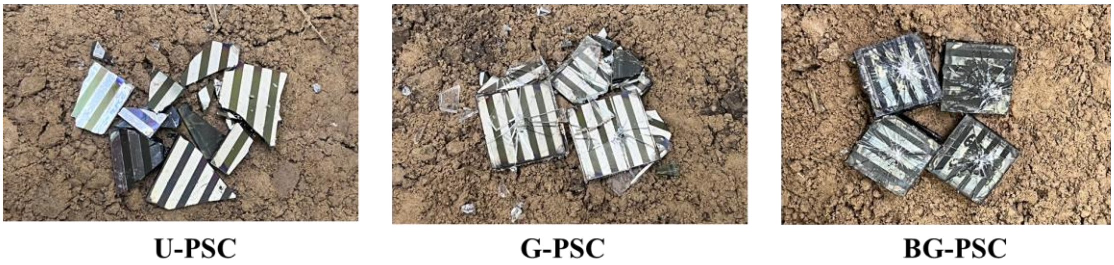

Supplementary Figure 21. Photographs of the test procedure for assessing the hazard of waste devices to the Yellow River soil. The devices damaged by the hail

crushing simulation were buried in the soil. Then DI water was poured daily at the location where the devices were buried. The soils below the buried devices were sampled on days 1, 7, 14, 21, and 30.

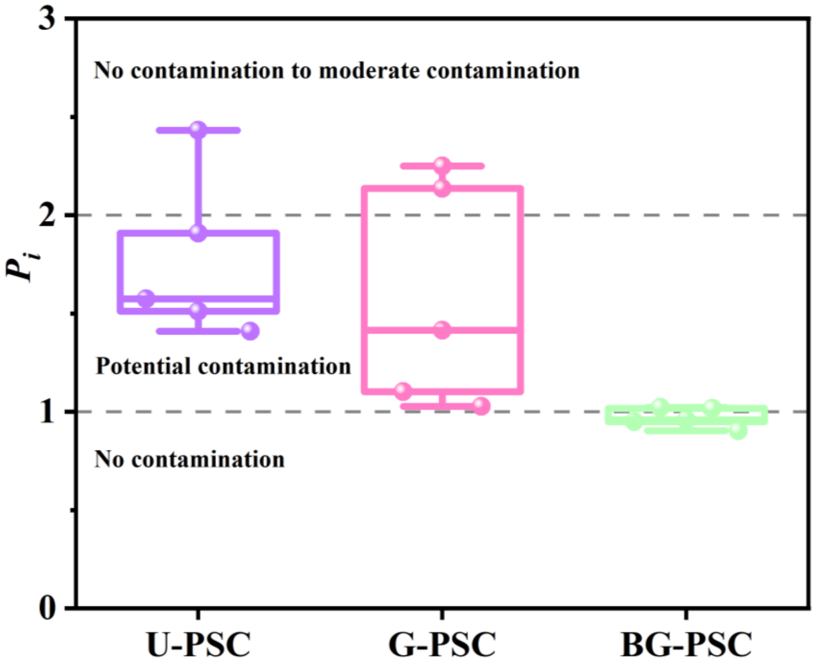

Supplementary Figure 22. Evaluation of Pb pollution of broken waste devices in the soil of the Yellow River. The evaluation result of single-factor pollution index technique (P_i). The Pb *Pi* range of U-PSC, G-PSC, and BG-PSC to the Yellow River soil are 1.40-2.43, 1.02-2.25, and 0.90-1.02 respectively. It can be noted that the contamination of the Yellow River soils by U-PSC and G-PSC are in a state of potential contamination and no contamination to moderate contamination, while the contamination of the Yellow River soil by BG-PSC is no contamination.

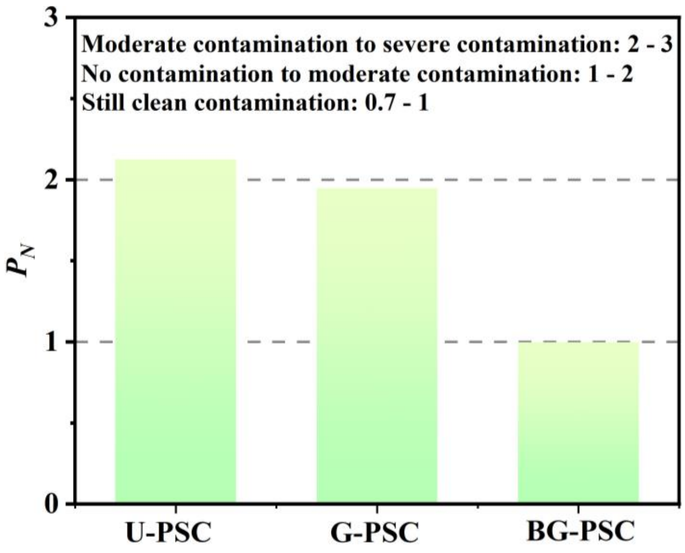

Supplementary Figure 23. Evaluation of Pb pollution of broken waste devices in the soil of the Yellow River. The evaluation result of Nemerow integrated pollution index (P_N). The P_N evaluation results shows that BG-PSC exhibits still clean contamination to the Yellow River soil compared with the pollution level of U-PSC and G-PSC to Yellow River soil are moderate contamination to severe contamination and no contamination to moderate contamination, respectively.

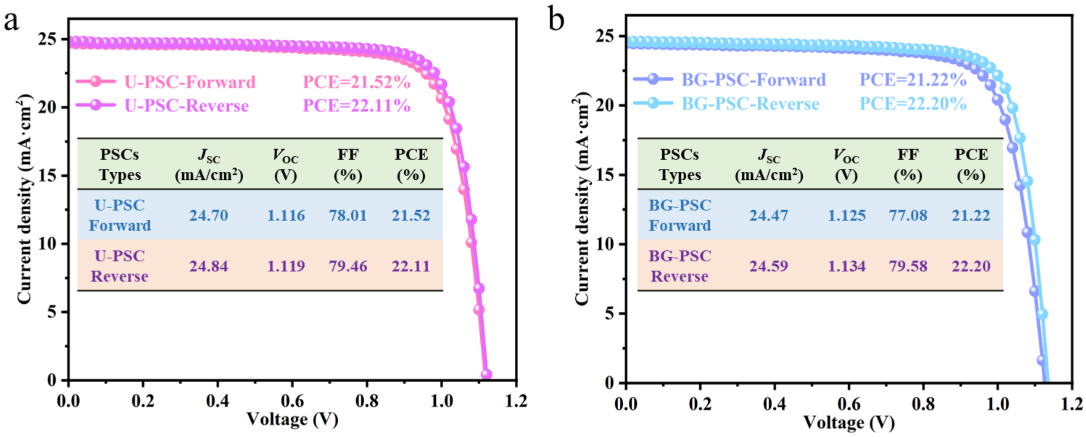

Supplementary Figure 24. Comparison of *J-V* curves before and after packaging devices. The *J*−*V* characteristics of U-PSC(a) and BG-PSC(b) under reverse or forward scanning direction

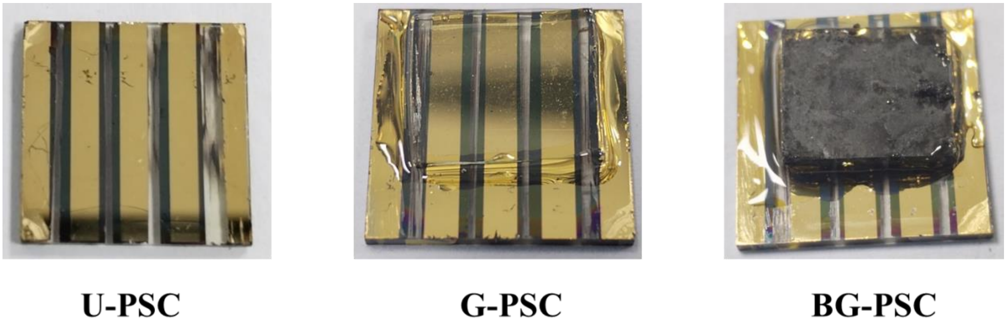

Supplementary Figure 25. Long-term stability test. Long-term stability test photographs of U-PSC, G-PSC and BG-PSC.

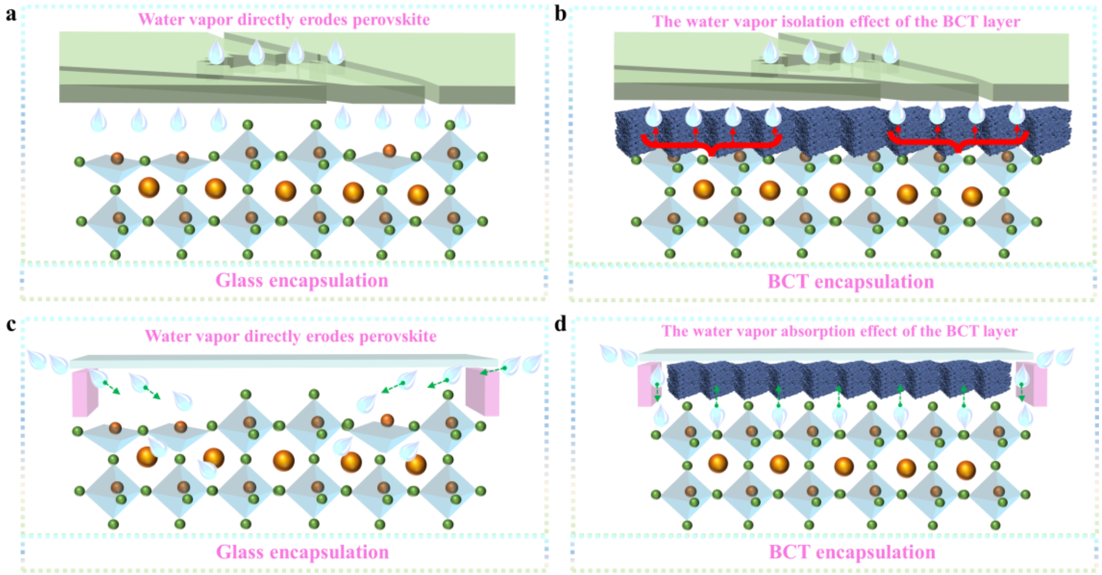

Supplementary Figure 26. The synergistic roles in terms of internal secondary water vapor adsorption and external water vapor isolation of the BCT. Comparison of water vapor isolation behavior between glass encapsulated PSCs (a) and BCT encapsulated PSCs (b) after crushing. Comparison of water vapor adsorption behavior between glass encapsulated PSCs (c) and BCT encapsulated PSCs (d) after the degradation of UV glue. In this work, the glass substrate with BCT protection layer was encapsulated onto the PSCs via sealing UV glue around the device edge, providing different roles in different scenarios. For example, when the PSCs encapsulated with

glass(G-PSC) are broken, the moisture in the humid environment will directly contact with the PSCs as the encapsulated glass substrate is broken, resulting in the rapid degradation of the perovskite light absorption layer, as shown in **Supplementary Figure 26a**. However, when PSCs encapsulated by BCT(BG-PSC) are broken, the BCT coating layer encapsulated on the device can form a thick water vapor barrier layer on the surface of the device because of its compactness. The barrier layer is able to isolate the water vapor from the perovskite layer when water vapor enters the device through the broken glass, thus isolating external water vapor, as shown in **Supplementary Figure 26b**. In addition, when the UV glue encapsulated at the edge of G-PSC is aged, water vapor can enter the device through the gap of the aged UV glue, leading to the degradation of the perovskite light-absorbing layer, as shown in **Supplementary Figure 26c**. However, when aging UV glue cracks appear in BG-PSC, the dense BCT coating layer can secondary adsorb internal water vapor which have entered the inner of the device via the gap of UV glue due to the unique porous structure of BCT, slowing down the erosion of perovskite layer by water vapor (**Supplementary Figure 26d**). Therefore, the BCT coating layer achieves the synergistic roles of secondary external water vapor isolation and water vapor adsorption based on water vapor isolation and absorption, improving the humidity stability of PSCs.

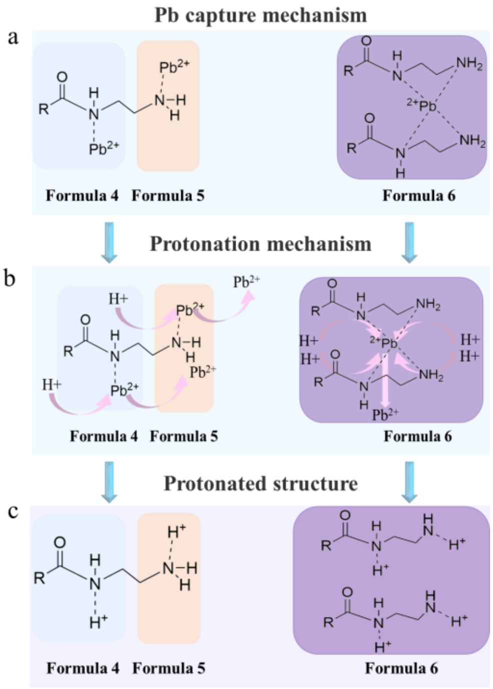

Supplementary Figure 27. The Pb adsorption and desorption mechanism of RCO-NH-R, RNH_2 and CONH-R-NH_2. a, Pb ions capture mechanism of RCO-NH-R, RNH_2 and CONH-R-NH_2. b, Protonation mechanism of RCO-NH-R, RNH_2 and CONH-R-NH_2. c, Protonated structure of RCO-NH-R, RNH_2 and CONH-R-NH_2.

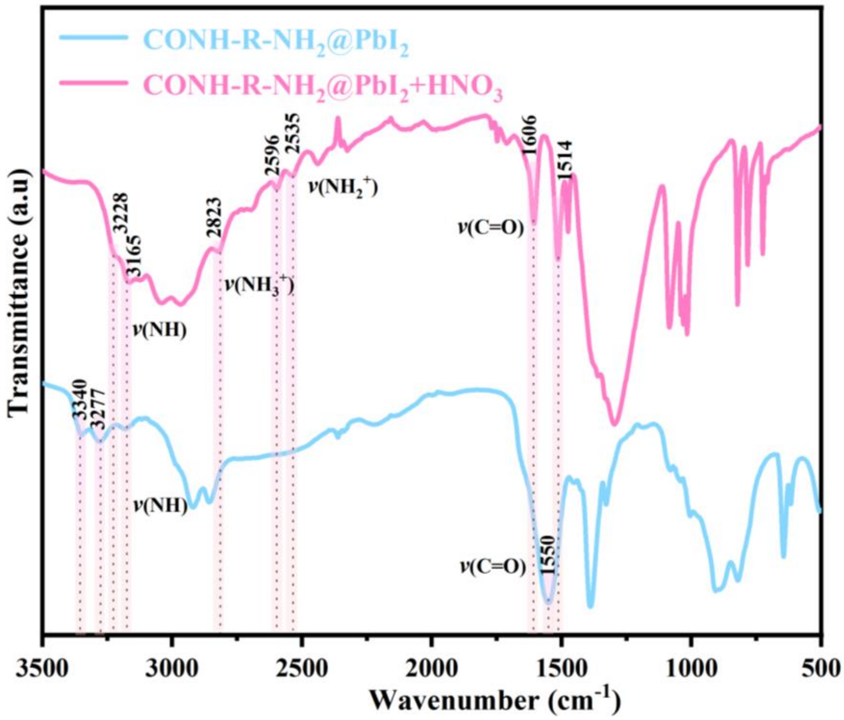

Supplementary Figure 28. The protonation mechanism proof of CONH-R-NH_2.

FTIR spectra of CONH-R-NH_2@ PbI_2 and CONH-R-NH_2@ PbI_2+ HNO_3

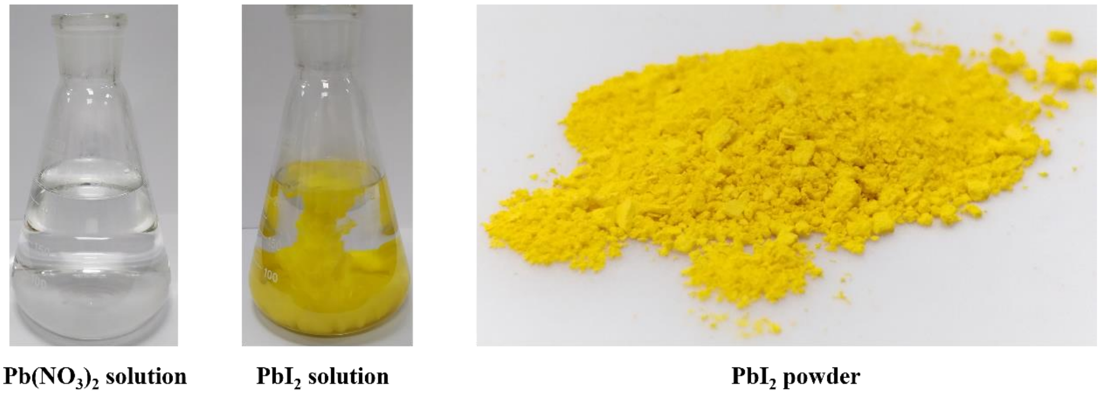

Supplementary Figure 29. Sustainable Pb management results of waste devices. Photographs of main recovery steps of yellow PbI_2.

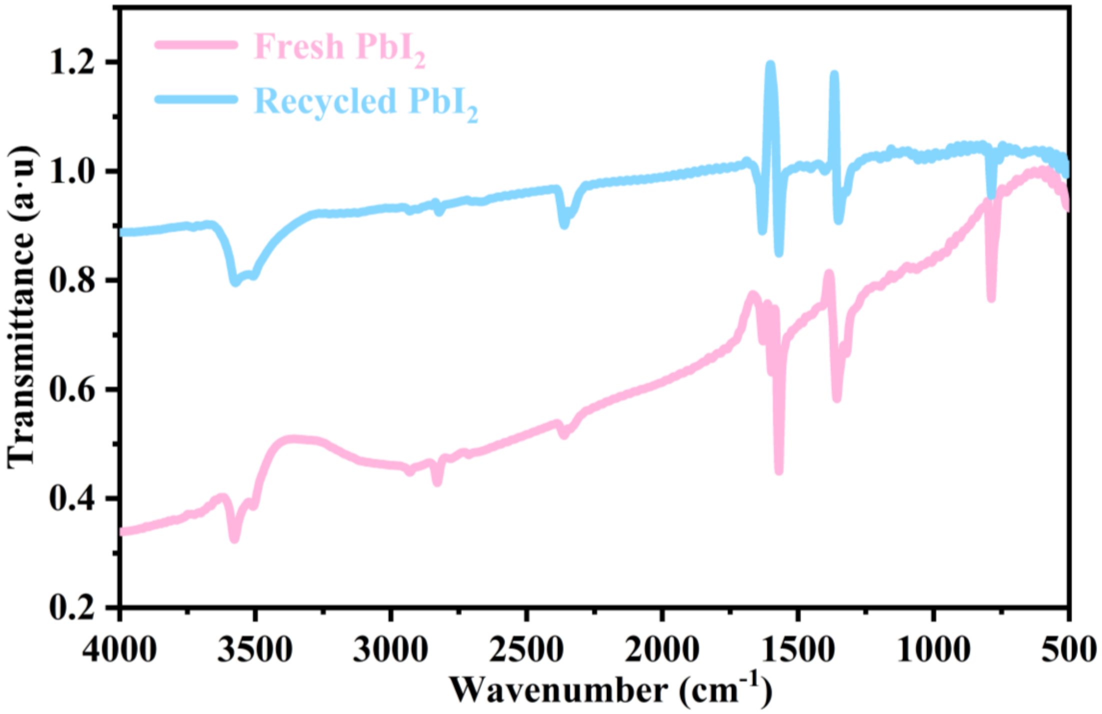

Supplementary Figure 30. The FTIR of commercial PbI_2 and recovered PbI_2. The recovered PbI_2 shows the same composition as commercial PbI_2, as can be verified via FTIR characterization, that the recovered PbI_2 exhibits consistently functional group stretching vibration peak compared with commercial PbI_2.

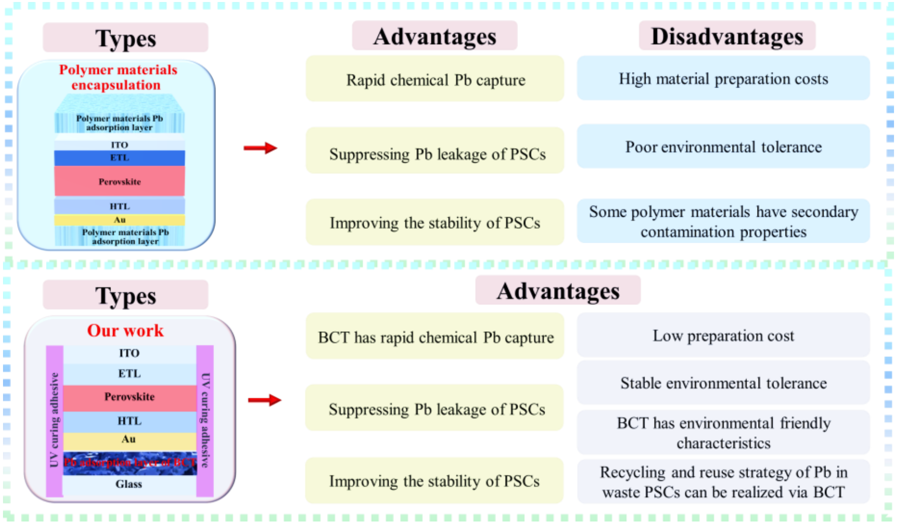

Supplementary Figure 31. The comprehensive comparison of polymeric material encapsulation and BCT encapsulation for reducing Pb contamination levels in PSCs. As shown in **Supplementary Figure 31**, our study focuses on the Pb sequestration of perovskite solar cells (PSCs) through the use of BCT materials as a sequestration agent. While there are stretchable and self-healable polymer materials available for this purpose, they have some drawbacks that may impact their suitability for large-scale commercialization.

Polymer materials offer rapid chemical Pb capture, suppress Pb leakage in PSCs, and improve their overall stability. However, they suffer from several significant disadvantages. Firstly, most organic polymer materials have high preparation costs, making them unsuitable for large-scale industrial usage. Secondly, these polymer materials often lack environmental tolerance and may fail due to factors such as lighting, high temperatures, and humidity when packaged behind the ITO/FTO substrate. This can lead to a loss of the Pb leakage inhibiting effect of PSCs. Thirdly, some polymer materials have secondary pollution characteristics that negatively impact the environmentally friendly profile of PSCs.

In contrast, BCT materials offer several advantages over polymer materials. Firstly, they have a low preparation cost, making them suitable for large-scale industrial usage, which reduces the overall cost of commercializing PSCs. Secondly, BCT materials have stable environmental tolerance, making them less susceptible to failure due to environmental factors such as lighting, high temperature, and humidity. Thirdly, BCT materials are environmentally friendly and do not have secondary pollution characteristics, unlike some polymer materials. Finally, BCT materials can recycle Pb in waste PSCs, which promotes the green commercialization of PSCs.

Overall, BCT materials offer an efficient way to reduce Pb contamination during the actual operating process of PSCs while reducing the cost of PSC production. They also offer a more environmentally friendly alternative to polymer materials and can promote the green commercialization of PSCs. Therefore, we believe that BCT materials are a promising alternative to polymer materials for sequestration of Pb in PSCs.

3. Supplementary Tables

Supplementary Table 1 Comprehensive comparison of the reported Pb adsorption materials.

Material	Encapsulation methods	Pb sequestration efficiency (%)	Environment of Pb leakage assessment	Pb recycling procedure	Recovery rate	Purity of recycled PbI_2	PCE of remanufactured PSCs and average SDF (%)	Cost ($ m^{-2})	Reference
P,P′-di(2-ethylhexyl)methanediphosphonic acid (DMDP) and N,N,N',N'-ethylenediaminetetrakis(methylenephosphonic acid)-poly(ethylene oxide)	Front + back encapsulation	96.00%	Lab	--	--	--	--	609.2	[4-6]
DMDP and ethylene vinyl acetate	Front + back encapsulation	99.90%	Lab	--	--	--	--	2935.10	[7]
Pb-adsorbing ionogel	Front + back encapsulation	99.90%	Lab	--	--	--	--	--	[8]
The mixture of	Front + back	99.73%	Lab	--	--	--	--	--	[9]

sulfonated graphene aerogel and polydimethylsiloxane	encapsulation								
Self-healing polymer	Front + back encapsulation	99.73%	Lab	--	--	--	--	12.30	[10]
Cation exchange resins (CER)	Back encapsulation	90.00%	Lab + real-world scenario(rainwater)	--	--	--	--	--	[11]
The mixture of a CER and an ultraviolet resin	Back encapsulation	90.00%	Lab	--	--	--	--	0.03	[12]
**Biomimetic “cage traps”**	**Back encapsulation**	**99.86%**	**Lab + real-world scenarios (Yellow River and soil)**	**Closed-loop Pb management procedure**	**96.07%**	**~99.99%**	**PCE=22.08%, average SDF=0.42%**	**0.39**	**This work**

Supplementary table 2 Comprehensive comparison and summary of Pb leakage rates of G-PSC and BG-PSC in deionized water and Yellow River water.

**Device types**	**Testing environment**	**Pb leakage rate (%)**	**Increasing amount of Pb leakage rate (%)**	**Pb sequestration efficiency (%)**
G-PSC	DI water	40.56	---	59.44
G-PSC	Yellow river	61.38	**20.82**	38.62
BG-PSC	DI water	0.14	---	99.86
BG-PSC	Yellow river	8.4	**8.26**	91.6

Supplementary table 3 The classification criteria of heavy metal pollution assessment in soils corresponding to I_{geo} values.

Evaluation methodology	Numerical range	Pollution level
Index geo-accumulation method (I_{geo})	$I_{geo} \leqslant 0$	no contamination
	$0 < I_{geo} \leqslant 1$	no contamination to moderate contamination
	$1 < I_{geo} \leqslant 2$	moderate contamination

Supplementary table 4 The classification criteria of heavy metal pollution assessment in soils corresponding to P_i values.

Evaluation methodology	Numerical range	Pollution level
Single-factor pollution index technique (P_i)	$P_i \leq 1$	no contamination
	$1 < P_i \leq 2$	potential contamination
	$2 < P_i \leq 3$	no contamination to moderate contamination

Supplementary table 5 The classification criteria of heavy metal pollution assessment in soils corresponding to P_N values.

Evaluation methodology	Numerical range	Pollution level
Nemerow integrated pollution index (P_N)	$0.7 < P_N \leq 1.0$	still clean contamination
	$1.0 < P_N \leq 2.0$	no contamination to moderate contamination

	$2.0 < P_N \leq 3.0$	moderate contamination to severe contamination

4. Supplementary References

1. Lin, Y., Fryxell, G. E., Wu, H. & Engelhard, M. Selective Sorption of Cesium Using Self-Assembled Monolayers on Mesoporous Supports. *Environ. Sci. Technol.* **35**, 3962-3966 (2001).
2. Tanaka, S., Doi, A., Nakatani, N., Katayama, Y. & Miyake, Y. Synthesis of ordered mesoporous carbon films, powders, and fibers by direct triblock-copolymer-templating method using an ethanol/water system. *Carbon* **47**, 2688-2698 (2009).
3. Hong, M. & Chen, E. Y. X. Future Directions for Sustainable Polymers. *Trends Chem.* **1**, 148-151(2019).
4. Li, X. et al. On-device lead sequestration for perovskite solar cells. *Nature* **578**, 555-558 (2020).
5. "Dichloromethylenediphosphonic acid disodium salt", https://www.sigmaaldrich.com/catalog/product/sigma/d4434?lang=en&region=HK.
6. "N,N,N',N'-Ethylenediaminetetrakis(methylenephosphonic Acid)", https://www.tcichemicals.com/US/en/p/E0393.
7. Li, X. et al. On-device lead-absorbing tapes for sustainable perovskite solar cells. *Nat. Sustain.* **4**,1038-1041 (2021).
8. Xiao, X. et al. Lead-adsorbing ionogel-based encapsulation for impact-resistant, stable, and lead-safe perovskite modules. *Sci. Adv.* **7**, eabi8249 (2021).
9. Li, Z. et al. Sulfonated Graphene Aerogels Enable Safe-to-Use Flexible Perovskite Solar Modules. *Adv. Energy Mater.* **12**, 2103236 (2022).
10. Jiang, Y. et al. Reduction of lead leakage from damaged lead halide perovskite solar modules using self-healing polymer-based encapsulation. *Nat. Energy* **4**, 585-593 (2019).
11. Chen, S. et al. Trapping lead in perovskite solar modules with abundant and low-cost cation-exchange resins. *Nat. Energy* **5**, 1003-1011 (2020).
12. Li, Z. et al. An effective and economical encapsulation method for trapping lead leakage in rigid and flexible perovskite photovoltaics. *Nano Energy* **93**, 106853 (2022).
