# Supplementary material for: Bioinspired “cage traps” for closed-loop lead management of perovskite solar cells under real-world contamination assessment
Source: Nat Commun. 2023 Aug 7;14:4730. doi: 10.1038/s41467-023-40421-8 (PMC10406821; doi:10.1038/s41467-023-40421-8)
Supplement: Supplementary file 3 — Description of Additional Supplementary Files [file 41467_2023_40421_MOESM3_ESM.pdf]

File Name: Supplementary Movie 1

Description: The main procedure of recycled PbI_2 precipitation. The $Pb(NO_3)_2$ solution was obtained by filtering the mixture of BCT and $Pb(NO_3)_2$. Firstly, 215 mL of $Pb(NO_3)_2$ solution was transferred to the conical flask. Then 1mol/L sodium iodide was added dropwise to the $Pb(NO_3)_2$ solution to form a yellow PbI_2 suspension.
