# Supplementary material for: Bioinspired “cage traps” for closed-loop lead management of perovskite solar cells under real-world contamination assessment
Source: Nat Commun. 2023 Aug 7;14:4730. doi: 10.1038/s41467-023-40421-8 (PMC10406821; doi:10.1038/s41467-023-40421-8)
Supplement: Supplementary file 5 — Reporting Summary [file 41467_2023_40421_MOESM5_ESM.pdf]

nature research

Corresponding author(s): Junjie Ma, Yiqiang Zhang, Yanlin Song

Solar Cells Reporting Summary

Nature Research wishes to improve the reproducibility of the work that we publish. This form is intended for publication with all accepted papers reporting the characterization of photovoltaic devices and provides structure for consistency and transparency in reporting. Some list items might not apply to an individual manuscript, but all fields must be completed for clarity.

For further information on Nature Research policies, including our data availability policy, see Authors & Referees.

Experimental design

Please check: are the following details reported in the manuscript?

1. Dimensions

Item	Yes/No	Details
Area of the tested solar cells	☒ Yes ☐ No	The device area is 0.04 cm^2.
Method used to determine the device area	☒ Yes ☐ No	The metal mask.

2. Current-voltage characterization

Item	Yes/No	Details
Current density-voltage (J-V) plots in both forward and backward direction	☒ Yes ☐ No	The (J-V) plots are provided in the manuscript.
Voltage scan conditions<br>*For instance: scan direction, speed, dwell times*	☒ Yes ☐ No	The bias ranges is from -0.1 to 1.2 V under reverse and forward voltage scan. Speed: 0.1 V/s, Dwell time: 10 ms.
Test environment<br>*For instance: characterization temperature, in air or in glove box*	☒ Yes ☐ No	All J-V results are measured at 25+2°C in glove box.
Protocol for preconditioning of the device before its characterization	☐ Yes ☒ No	No precondition is used in this work.
Stability of the J-V characteristic<br>*Verified with time evolution of the maximum power point or with the photocurrent at maximum power point; see ref. 7 for details.*	☐ Yes ☒ No	We provided the stability of PSCs under continuous light at AM1.5G, 100 mW cm^-2 with 60±5°C in Fig.4e.

3. Hysteresis or any other unusual behaviour

Item	Yes/No	Details
Description of the unusual behaviour observed during the characterization	☒ Yes ☐ No	Very minor hysteresis for the devices.
Related experimental data	☒ Yes ☐ No	The hysteresis effect is not the research priority in this work, but we have provided J-V plots in both forward and backward direction in supplementary material.

4. Efficiency

Item	Yes/No	Details
External quantum efficiency (EQE) or incident photons to current efficiency (IPCE)	☒ Yes ☐ No	EQE are provided in Fig. 4d in the manuscript.
A comparison between the integrated response under the standard reference spectrum and the response measure under the simulator	☒ Yes ☐ No	EQE spectra demonstrated matchable integrated JSC values to J-V scan data.
For tandem solar cells, the bias illumination and bias voltage used for each subcell	☐ Yes ☒ No	Tandem solar cells are not covered in this paper.

5. Calibration

Item	Yes/No	Details
Light source and reference cell or sensor used for the characterization	☒ Yes ☐ No	The J-V curves were obtained using a B2901A source meter equipped with a solar simulator (Enlitech, SS-X50) under simulated sun illumination (AM 1.5G, 1 sun).The AM 1.5G illumination was calibrated by standard Si solar cell.
Confirmation that the reference cell was calibrated and certified	☒ Yes ☐ No	The standard silicon solar cell calibrated by Newport was used to calibrate the light intensity to AM1.5G one sun (100 mW cm^-2).

Calculation of spectral mismatch between the reference cell and the devices under test

[x] Yes
[ ] No

The standard silicon solar cell calibrated by Newport was used to calibrate the light intensity to AM1.5G one sun (100 mW cm^-2).

6. Mask/aperture

Size of the mask/aperture used during testing

[x] Yes
[ ] No

Metal mask with area of 0.04 cm^2.

Variation of the measured short-circuit current density with the mask/aperture area

[x] Yes
[ ] No

All J-V results are measured with mask.

7. Performance certification

Identity of the independent certification laboratory that confirmed the photovoltaic performance

[ ] Yes
[x] No

The photoelectric conversion efficiency is not measured by independent certification laboratory.

A copy of any certificate(s)
Provide in Supplementary Information

[ ] Yes
[x] No

The photoelectric conversion efficiency is tested under standard test conditions in our laboratory.

8. Statistics

Number of solar cells tested

[x] Yes
[ ] No

27 solar cells were tested.

Statistical analysis of the device performance

[x] Yes
[ ] No

These have been stated in the manuscript in Fig. 4c and Fig.5 g.

9. Long-term stability analysis

Type of analysis, bias conditions and environmental conditions
For instance: illumination type, temperature, atmosphere humidity, encapsulation method, preconditioning temperature

[x] Yes
[ ] No

Fig. 4e. The encapsulated devices stability is aged under continuous light at AM1.5G, 100 mW cm^-2 with 60±5°C in16-channel solar cell stability test system. Fig.4f and Fig.4g. The encapsulated devices stabilities are aged under 25°C, 50% and 25°C, 85% relative humidity conditions in a constant temperature and humidity chamber.
